# Tritrophic Interactions among Arthropod Natural Enemies, Herbivores and Plants Considering Volatile Blends at Different Scale Levels

**DOI:** 10.3390/cells12020251

**Published:** 2023-01-07

**Authors:** Muhammad Yasir Ali, Tayyaba Naseem, Jarmo K. Holopainen, Tongxian Liu, Jinping Zhang, Feng Zhang

**Affiliations:** 1 MARA-CABI Joint Laboratory for Bio-Safety, Institute of Plant Protection, Chinese Academy of Agricultural Sciences, Beijing 100193, China; 2 Key Laboratory of Insect Ecology and Molecular Biology, College of Plant Health and Medicine, Qingdao Agricultural University, Qingdao 266109, China; 3 State Key Laboratory for Biology of Plant Diseases and Insect Pests, Institute of Plant Protection, Chinese Academy of Agricultural Sciences, Beijing 100193, China; 4 CABI East & South-East Asia, Beijing 100081, China; 5 Department of Botany, University of Agriculture Faisalabad, Faisalabad 38000, Pakistan; 6 Department of Environmental Science, University of Eastern Finland, 77100 Kuopio, Finland

**Keywords:** biological control, indirect defense, insect herbivores, natural enemies, plant volatiles

## Abstract

Herbivore-induced plant volatiles (HIPVs) are released by plants upon damaged or disturbance by phytophagous insects. Plants emit HIPV signals not merely in reaction to tissue damage, but also in response to herbivore salivary secretions, oviposition, and excrement. Although certain volatile chemicals are retained in plant tissues and released rapidly upon damaged, others are synthesized de novo in response to herbivore feeding and emitted not only from damaged tissue but also from nearby by undamaged leaves. HIPVs can be used by predators and parasitoids to locate herbivores at different spatial scales. The HIPV-emitting spatial pattern is dynamic and heterogeneous in nature and influenced by the concentration, chemical makeup, breakdown of the emitted mixes and environmental elements (e.g., turbulence, wind and vegetation) which affect the foraging of biocontrol agents. In addition, sensory capability to detect volatiles and the physical ability to move towards the source were also different between natural enemy individuals. The impacts of HIPVs on arthropod natural enemies have been partially studied at spatial scales, that is why the functions of HIPVs is still subject under much debate. In this review, we summarized the current knowledge and loopholes regarding the role of HIPVs in tritrophic interactions at multiple scale levels. Therefore, we contend that closing these loopholes will make it much easier to use HIPVs for sustainable pest management in agriculture.

## 1. Introduction

Volatile organic compounds (VOCs) are released by the majority of vascular plants on a constant basis; however, under biotic and abiotic stress, emissions may significantly increase and change [1]. Herbivore insects feeding on plants caused the emission of novel volatile chemicals, also known as herbivore-induced plant volatiles (HIPVs) [2] that attract natural enemies of the herbivore insects such as predators and parasitoids [3]. This was initially demonstrated in pioneering investigations using predatory and spider mites in 1988 by Dicke & Sabelis [4] and it was eventually shown to be a more universal phenomena involving multiple plant species, herbivores and predator/parasitoid wasps in 1990 by Turlings and his colleague [5].

HIPVs are thought to enhance the emitting plant’s fitness either directly or indirectly [6,7]. Direct defense slows down the herbivore’s rate of consumption or discourages it from approaching and attacking [8]. For instance, females of the defoliating moth, *Tortrixvirid ian* L. (Lepidoptera: Tortricidae) avoided English oak tree, *Quercus robur* L. (Fagaceae: Fagales), with an herbivore resistant phenotype because they released HIPVs that contained the sesquiterpenes *α*-farnesene and germacrene-D [9]. Trees generating other common HIPVs, such as homoterpene (*E*)-4,8-dimethyl-1,3,7-nonatriene (DMNT) and monoterpene *β*-ocimene, were susceptible and largely defoliated in the same outbreak region [9]. On the other hand, indirect defense entails the recruitment of herbivores’ natural enemies which cause predation or parasitization of hosts and thus lessen the plant damage. Natural enemies can use these HIPVs emissions to find herbivore-infested plants, which act as long-range kairomones [10,11,12,13]. One innovative strategy for achieving effective biological control has been the use of HIPVs to entice natural enemies [14,15,16,17].

HIPVs are released by both above and below ground plant parts [18] and can be synthesized by many plant species [19]. Herbivores that feed on the roots of plants release HIPVs, which serve as underground attractants for parasitic nematodes [20,21]. Additionally, there are systemic effects of above-ground herbivory on below-ground HIPV production and vice versa [22]. The Scots pine, *Pinus sylvestris* L. (Pinales: Pinaceae) was defoliated by diprionid sawflies including *Pikonema alaskensis* Rohwer (Hymenoptera: Diprionidae), which induced considerable HIPVs emissions from the shoots but significantly lessened sesquiterpene and monoterpene emissions from the root system [23]. This was predicted to be connected to a decreased allocation of carbon to subterranean regions following defoliation. Arbuscular mycorrhizal infection of bean plant roots changed the HIPVs composition produced by the leaves, making the foliage less appetizing to predators [24], while the terpene pool of pine needles was unaffected by an ectomycorrhizal root symbiont [25]. These studies emphasize the intricate and systemic character of HIPVs and call for a comprehensive understanding of volatile emissions from a plant and their multiple related functions.

HIPVs operating as attractants is still a speculation and has become reality as the number of field trials examining HIPV-mediated attraction and its ramifications for pest reduction has risen considerably over the past 10 years. Current research have given an overall picture of recent outdoor works to augment biocontrol enemy populations using HIPVs, with highlighting on those research exploiting synthetic compounds in controlled-release dispensers and figure out a catalog for upcoming research needs. Specifically, recent HIPVs reviews discuss: (i) functional changes in insects’ natural enemies; [3] (ii) HIPVs under air pressure; [26] (iii) molecular mechanism in HIPV signaling; [27] (iv) non-target effects of HIPVs [14]. However, sufficient progress has been made since then in understanding the informational content of volatile mixes. It is usually claimed that the volatile mixture’s composition could give antagonists precise information about the types of herbivores present, as well as their age, developmental stage, and quantity. However, it is difficult to prove this potential.

In this review, we concentrate on the various functions and outcomes of inducible VOC molecules following their release from the VOC synthesis plant, considering biological, chemical, and physical elements. In view of the most recent findings on arthropod natural enemies’ behavior and insect olfaction, we investigate the specificity of herbivore-induced plant volatiles as signals for herbivore natural enemies at multiple scale levels. First, we outline the origins of HIPVs in tritrophic systems and discuss how arthropod natural enemies might use these kairomones to their advantage while searching for hosts or prey. We will also focus on the biological and ecological effects of post-emission VOCs and the byproducts of their atmospheric interaction. We also review how plant fitness may be enhanced by the atmospheric breakdown of released VOCs. We conclude by highlighting potential improvements to the use of HIPV-based lures for attracting sufficient natural enemies to reduce damaging insect pest populations and crop losses to economically acceptable levels, whilst simultaneously boosting field crop yields.

## 2. Chemical Footprints of Plant Volatiles

Plant priming is a phase of sensitization that leads to a stronger and quicker induced defence response following subsequent herbivore attack compared to a non-primed one [28]. Priming decreases the induced defense response’s time lag and may result in a greater reaction, generally at a cheaper cost to the plant [29]. Defense priming can occur following stimulation to induced plant volatiles from neighboring plants, exposure to other (synthetic) elicitors such as beta-aminobutyric acid (BABA), or the addition of rhizobacteria [28,30,31,32]. For instance, the volatiles emitted from leaves that have been damaged by herbivores can stimulate the secretion of extrafloral nectar in lima beans [33]. In response to feeding by a lepidopteran herbivore, maize plants exposed to volatiles of damaged maize seedlings released more parasitoid-attracting sesquiterpenes than unprimed plants. Volatiles may potentially enable ‘eavesdropping’ between several plant species, which would directly increase defenses. This is true with wild tobacco plants which strengthen their defenses and develop greater herbivore resistance after being exposed to volatiles released by sagebrush damage. Lastly, while BABA-mediated priming is particularly effective against diseases, there is evidence that it can also inhibit aphid development without directly affecting the aphid’s parasitoids [34]. This shows that plant defense primers like BABA could be used in IPM tactics.

**Figure 1:**
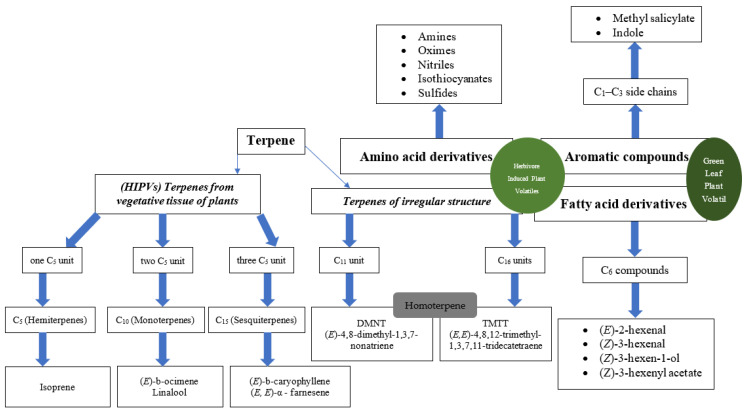
Plant volatile organic compounds (VOCs) consist of chemicals from different chemical classes, which have been identified in blends after herbivore damage.

A huge variety of chemicals emitted from plants have been recorded in volatile combinations [35,36]. It is easy to envision that each plant species may produce a unique mixture of volatile compounds that would enable herbivores and their biocontrol agents to identify particular plant species. The principal herbivore-induced volatiles, on the other hand, reveal that most plant species produce the same or similar constituents, regardless of their taxonomic affinities (Box 1). Examples include the sesquiterpenes (*E*)-β-caryophyllene and (*E*, *E*)-α-farnesene, the C_11_ homoterpene DMNT, and the fatty acid derivatives known as green leaf volatiles (GLVs), including (*Z*)-3-hexen-1-ol and (*Z*)-3-hexenyl acetate, which are frequently present in volatile mixtures generated by a variety of plant types after herbivore damage (Figure 1) [37,38,39,40,41]. Apart from herbivore damage, a clear difference in novel volatile organic compounds (VOC) emission were noted before and after fruit fermentation process which are important in host location for *Drosophila* spp [42]. The spotted-wing drosophila, *Drosophila suzukii* Matsumura (Diptera: Drosophilidae), is attracted to various VOCs released from different small fruit crops at ripening stage, but β-cyclocitral terpenoid in the strawberry leaf is studied to be very attractive towards *D. suzukii* (however not to all *Drosophila* spp.) [43]. Despite this, there are significant variances in the relative abundances of these chemicals between species, and less common compounds frequently exhibit a wide range of variations that may contribute to specificity. These variations might make it easier to identify different species if they are seen by herbivore adversaries. As reported, plant volatile emission within species can change depending on the herbivore presence [44].

**Figure 2:**
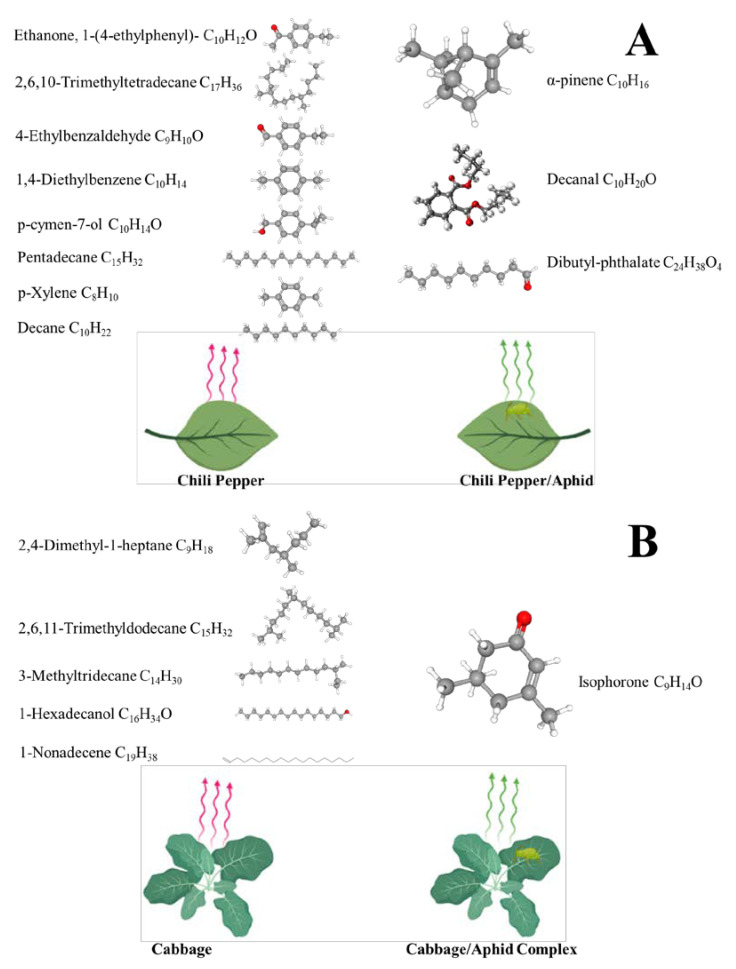
A schematic diagram representing the emission of compounds before and after aphid (*Myzus persicae*) damage on chili pepper (**A**), and cabbage (**B**), plants. The volatiles which emit before herbivore attack act as attractant in case of chili pepper while repellent in cabbage. Volatiles from both aphids fed plants (HIPVs) act as attractant though the compounds are different [7].

Insects and their natural enemies exhibit a positive or negative response to constitutive plant VOC chemicals which act as attractant [45] or repellent [46], a recent study revealed that the volatiles emitted from chili pepper act as attractant [47,48,49,50,51,52,53,54] whereas volatiles from cabbage plant act as repellent towards parasitoids (Figure 2) [55,56,57,58,59]. Furthermore, parasitoid wasps show significantly stronger response to aphid-induced VOCs in comparison with plant VOCs to the same species due to the presence of novel compounds which emitted after herbivore damage [60,61,62,63]. The response of the parasitoid *A. varipes* towards phthalic acid demonstrate that phthalic acid derivatives emit from *M. persicae* fed chili pepper act as attractant [7]; however, studies on phthalic acid are very limited so it needs to consider for further studies including lab and field bioassays.

Box 1 **Plant volatiles linked to the attraction of herbivore antagonists**

Plants produce a wide variety of volatile metabolites due to their high vapor pressure under normal circumstances. Over 1700 volatiles, aside from simple gases like ethylene, O2, CO2, and water vapor, are reported to be released by plants [44]; however, only a small portion of them are released by specific plants as a result of herbivore damage. They can be categorized into four groups.

**Terpenes**, the biggest group of plant volatiles, are categorized according to the number of branching C5 units they have in their structural makeup. Two terpenes that are frequently found following herbivore damage have irregular structural makeup.

A substantial family of volatile derivatives is established as a result of the oxidation of **fatty acid derivatives**. Following herbivore damage, subsequent lipoxygenase and hydroperoxide lyase action produces C6 compounds known as GLVs because they emit the distinctive odor of green leaves. In contrast to tryptophan biosynthesis, which produces indole derivatives, the metabolism of **aromatic substances** produces a series of molecules with simple aromatic rings and C1-C3 side chains. Methyl salicylate and indole are the most significant members of this category following herbivore damage. Several **amino acid derivatives** made from amino acids are released upon herbivore damage. These substances may be more prevalent than is currently believed because they are frequently less successfully recovered in routine headspace samples than terpenes, GLVs, and aromatics. There are several excellent references available for chemical structures and additional details on the chemistry and biochemistry of herbivore-induced volatiles (Figure 1) [3,64,65].

Natural enemies can learn important information about the types of hosts or prey that are present on a plant and their feeding guilds from variations in the contents and proportions of the constituents among herbivore volatile emissions [66,67,68,69]. For example, the volatile mixture emitted when turnip rape, *Brassica rapa* L. (Lepidoptera: Brassicaceae), are attacked above ground by large cabbage white butterfly, *Pieris brassicae* L. (Lepidoptera: Pieridae) differs greatly from the volatile mixture released when the plants are fed by the root herbivore cabbage root fly, *Delia radicum* L. (Diptera: Anthomyiidae) [70]. Salicylaldehyde and 4-methyltridecane predominate in the volatile mixture of plants damaged by *D. radicum*, but methyl salicylate is specific for cabbage damaged by *P. brassicae* [70]. The leaf beetle, *Chrysomela lapponica* L. (Coleoptera: Chrysomelidae), initially utilizes the salicyl glucosides (SGs) of its host plants to sequester salicylaldehyde, which acts as a defense for generalist natural enemies; however, attracts specialist natural enemies [71]. A predator fly larva, *Parasyrphus nigritarsis* Zetterstedt (Diptera: Syrphidae), and parasitoids, phorid flies, *Megaselia* spp.; (Diptera: Phoridae) were attracted to larval secretions reared on SG-rich and SG-poor hosts [72]. In a field study, sticky card traps were used for monitoring within methyl salicylate treated and untreated plots and significantly more syrphid flies (Diptera: Syrphidae) and green lacewings (Neuroptera: Chrysopidae) were collected on traps near to the methyl salicylate lure, but there were no variations in abundance at traps 1.5 m from the attraction [73].

The GLV hexyl acetate is emitted in significant relative levels when roots and shoots are targeted concurrently [70]. Specific elicitors in oral secretions used throughout the feeding process may be the origin of the varied profiles produced by different herbivores. A number of new elicitors linked to herbivore feeding and oviposition have currently been identified, in addition to the well-known fatty acid-amino acid conjugates and *β*-glucosidases in the lepidopteran larval oral secretions [74,75,76,77]. The elicitors now known, however, do not appear to be sufficient to account for the majority of the variations in plant volatile emission patterns. Different defense-signaling pathways and accompanying phytohormones may be differentially induced, which could explain the specificity in elicitor detection [78].

Jasmonic acid-dependent signaling is typically activated by insects that feed on leaves, while salicylic acid-dependent signaling is occasionally antagonistic to jasmonic acid signaling and is induced by phloem feeding and in response to viral infections too [44,79]. To understand what drives specialization in plant volatile emission, previous reviews have been made on functions, biosynthesis and metabolic engineering of plant VOCs [80,81].

Plants attacked by several developmental stages of the identical insect herbivore species provide more proof of the selectivity in volatile emission [82,83]. For example, larvae of the willow leaf beetle, *Plagiodera versicolora* Laicharting (Coleoptera: Chrysomelidae) trigger young wolly pod willow, *Salix eriocarpa* Franch. & Sav. (Malpighiales: Salicaceae) trees to emit 6 out of 17 detected volatile chemicals in substantially larger proportions as compared with after adult beetle herbivory. The overall emission rates from larval feeding are larger than those from adult beetle damage [82]. Egg deposition has also been claimed to influence volatile blends [76,84,85], with egg-induced volatile mixtures different from those induced by larval feeding [34]. Because the rate of emission of particular compounds is frequently strongly connected with the degree of inflicted damage, the volatile mixture emitted from plants could also provide useful insight on the number of herbivores currently feeding on a plant [85,86,87]. Even whether or not herbivores have already been attacked by parasitoids, as well as the species that attacked them, can be determined by changes in volatile emissions [88], which could include important information for other parasitoids.

**Figure 3:**
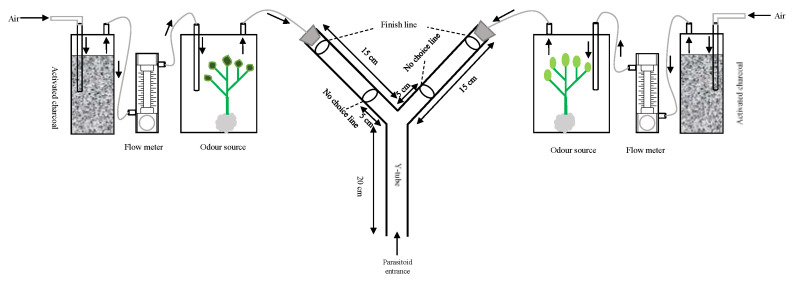
Schematic diagram of the Y-tube olfactometer used in our latest study Ali et al. 2022 [7].

**Figure 4:**
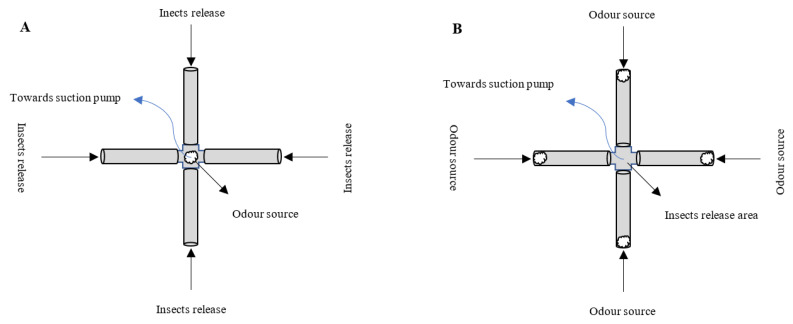
Schematic diagram of the four-arm olfactometers, (**A**): 1 odor source testing with 4 different wasps’ treatments, (**B**): 4 separate odor sources testing with same wasps.

Research on volatiles emission, odor trapping and insect behavior are very hot currently, and olfactometers are commonly used devices now a days to carry out these studies, and one of the earliest and best descriptions of an olfactometer comes from (McIndoo 1926) [89]; see also Snapp & Swingle (1929) [90]. He investigated the attractiveness of host-plant odors to beetles by placing the insects in the base of a Y-shaped glass tube and exposing them to odors introduced through the tube’s two arms (Figure 3). An insect that crawled into one of the arms was supposed to favor the odor provided through that arm. Y-tube olfactometers or T-tube olfactometers [91], are yet frequently utilized to investigate the olfactory responses of several arthropods, predatory spider mite, *Phytoseiulus persimilis* (Mesostigmata: Phytoseiidae) [92], white butterfly parasitoid, *Cotesia glomerate* (Hymenoptera: Braconidae) [93], cabbage seed weevil, *Ceutorhynchus assimilis* (Coleoptera: Curculionidae) [94], phytophagous mites, *Tetranychus urticae* (Mesostigmata: Tetranychidae) [95], corn leaf aphid, *Rhopalosiphum maidis* (Hemiptera: Aphididae) [96], bark beetle parasitoid *Roptrocews xylophugorum* (Hymenoptera: Pteromalidae) [97] and many more. The four-arm olfactometer was developed by Pettersson in 1970 [97], and Vet et al. (1983) further described and improved it [98]. Odor sources are placed in the center and insects are released from the tubes entrance (Figure 4A), insects are placed into a chamber that has been built with four different odor fields in this olfactometer and let insects choose (Figure 4B). Principally, four separate odor sources can be examined; however, it normally depends upon the hypothesis of study having more than two odor source or insects. Four-arm olfactometers are more beneficial for studies relevant with direct behavioral observations having several treatments.

The pool of odorant receptors (ORs) and odorant binding proteins (OBPs) expressed in the olfactory organs of a particular insect species first encodes the stimulus quality of the volatiles [98]. While OBPs are usually believed to be crucial for the solubilization and transportation of odorants [99], and the ORs are molecular actors that trigger the olfactory signaling cascade. Despite this, there are a number of studies in which different herbivore species, developmental stages, feeding guilds and number of attackers were discovered not to change volatile emissions of their host plants significantly [2,100,101]. For example, the spectrum of coyote tobacco, *Nicotiana attenuate* Torr. ex S. Watson (Solanales: Solanaceae) volatiles induced by herbivory, five-spotted hawkmoth, *Manduca quinquemaculata* Haworth (Lepidoptera: Sphingidae), tobacco flea beetle, *Epitrix hirtipennis* Melsheimer (Coleoptera: Chrysomelidae) and a suck fly, *Tupiocoris notatus* Distant (Hemiptera: Miridae) is similar to the compounds emitted in only moderately different proportions, specifically three volatiles *cis*-3-hexen-1-ol, *cis*-*a*-bergamotene and linalool which enhanced the predation rate of the big-eyed bug predator, *Geocoris pallens* Say (Heteroptera: Geocoridae) up to 90% [101]. Experimental techniques must be developed in order to determine whether herbivore-induced emission is actually specifically enough to be sensed by insect predators and parasitoids. Because the number of individual compounds may not be associated with their informational worth, a more extensive chemical analysis of plant volatile blends is required that involves even fewer common chemicals.

Additionally, explicit statistical methods are also required to determine whether blends differ considerably. These must be considered the simultaneous changes in composition and abundance, the possibility of autocorrelation among substances generated from the same biosynthetic route, and the fact that data are frequently asymmetrically allocated and heteroscedastic [102]. A significant improvement in studying induced volatile emission would result from collecting samples in the field to determine how blend compositions are changed by the normal biotic and abiotic variables that exist there. The majority of herbivore-induced volatile collection experiments have been conducted in the controlled environments of a laboratory or greenhouse, where the proof of specificity is merely inferential. Ultimately, evidence shows that the specificity of HIPVs (for respondents) is conferred by the volatile blend and the proportion of its constituents. The following sections focus on herbivore natural enemies’ behavior and their consciousness of plant volatiles.

## 3. Different Scales of Interaction

### 3.1. The Spatial and Temporal Scales of Parasitoid Interactions with Plant

**Figure 5:**
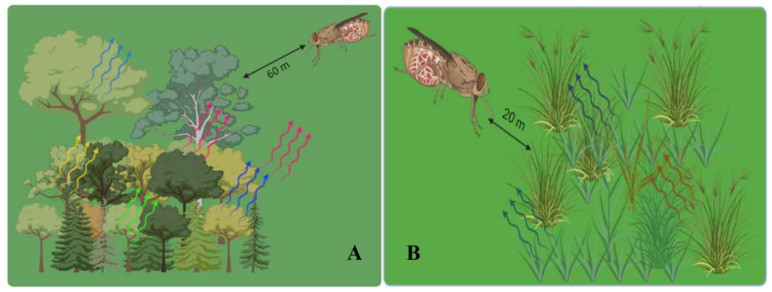
The response of tsetse flies to host odors. (**A**): from larger distances (60 m) in woodlands; (**B**): from close distance (20 m) in open fields [106].

**Figure 6:**
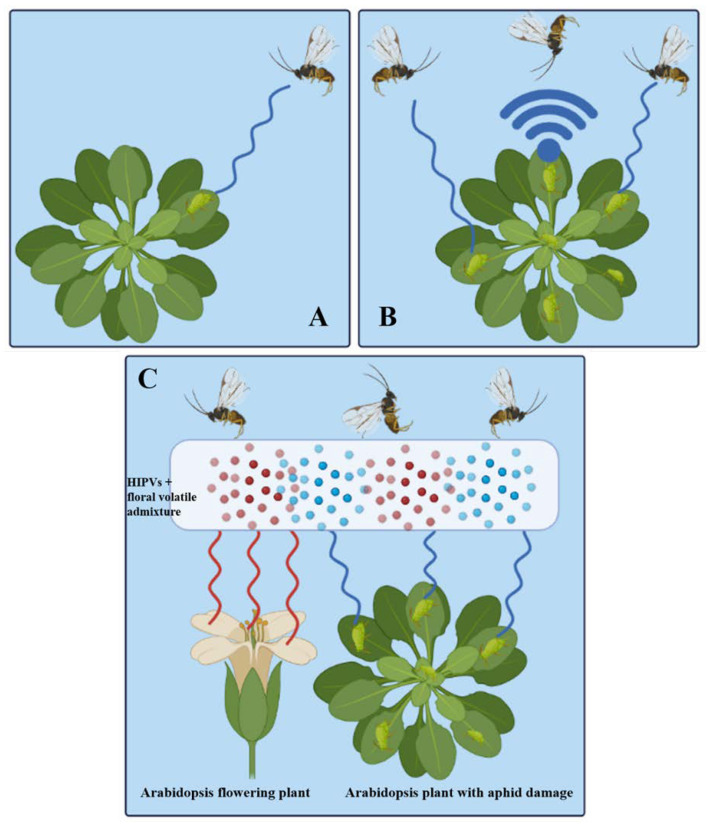
The response of parasitoid wasp *Diaeretiella rapae* Mc’Intosh (Hymenoptera: Braconidae) to host odors. (**A**): less attractive when aphid (*M. persicae*) damage was low; (**B**): attraction gradually increase as the damage of the aphid increase due to the emission of more quantity of HIPVs; (**C**): admixture of volatiles from flowers and aphid (HIPVs) in the air disturbed parasitoid’s host location and start repelling parasitoid wasps [109].

A female parasitoid has a short window of opportunity after emerging from her cocoon to scout their surroundings and learn about the quality of the patch [103]. When hosts are dispersed in different directions, perceptual range which is a product of perception sensitivity and scent dispersion will affect host detection [104]. The smell perception range of parasitoids in field settings is not well understood, nor is it known whether this range varies between species. Depending on the quantity of scent sources, several investigations using synthetic volatile sources and moths exhibit antennal reflexis to odor sources in the field up to 60 m away from the odor sources [105]. The landscape, which affects how far odors move, also affects the distance over which they are sensed. For instance, tsetse flies react to host odors from significantly greater distances (60 m) in woodlands (Figure 5A) than in open fields (20 m) (Figure 5B), demonstrating that these vegetative structures allow odor plumes to remain intact longer [106]. The admixture of the *Arabidopsis thaliana* L. (wild-type, Columbia-0) (Brassicales: Brassicaceae) flower plants and herbivores-damaged plants are likely to communicate with each other in the atmosphere, adding to the complexity of the natural enemies’ olfactory world (Figure 6) [107,108]. Floral scents diminished the attractiveness of herbivore-infested plants to parasitoids by 43.5% and four of the five parasitoid species evaluated were impacted [109]. Research with the white butterfly parasitoid, *Cotesia glomerate* L. (Hymenoptera: Braconidae) found that the impacts of floral scents are dose-dependent, and that floral odors were less disruptive in a wind tunnel than in an olfactometer [109]. Floral odors can function as background ‘noise’ reducing the appeal of chemical mixtures utilized by natural enemies to locate their hosts [110]. The quantity and quality of odors released by plant species employed in flower strips used in Desurmont’s study [109], as well as their concentration and proximity with pest-infested plants must be taken in account to secure the potency of conservation biological control strategy.

HIPV compounds often have brief air half-lives after being released by plants, which may reduce their ability to draw herbivore natural enemies and influence other ecological interactions [111,112]. The differential half-lives of these compounds could indicate to predators and parasitoids the ‘freshness’ of the signal, and so help them choose between competing signals. Different weather conditions, especially in the presence of oxidants, affect perceptual range by influencing odor plume movement (Table 1) [113]. While plants can communicate information about herbivore attacks [5,114], parasitoids’ detection and processing of these cues may vary depending on how close they are to the HIPV source, even though empirical evidence for this is missing yet [115]. Based on the spatial scale, a number of elements may be of dominant interest. Interestingly, reactive VOCs play a variety of roles in atmospheric processes, including the generation of ozone in NOx-polluted atmospheres [116], formation of OH-radicals [117], formation of organic nitrates [118] and formation of secondary aerosols (SOA) and photochemical smog [117,119,120].

Herbivore-damaged plants release a mixture of volatiles that is quantitatively and/or qualitatively different from the blend released when the plant is not damaged or mechanically damaged [122,122]. As a result, host herbivore-damaged plants are more likely to be parasitized than healthy or mechanically harmed plants [123,124]. The severity of herbivore load and herbivore damage is positively associated with HIPV emission [125] and, consequently, extremely infested plants are significantly more attractive to parasitoids [87]. Phloem feeding herbivores generally induce lower amounts of volatiles compared with chewing herbivores [126], perhaps due to the minor tissue damage induced by phloem feeders. In addition to influencing a parasitoid’s initial attraction to a plant, HIPVs can also stimulate parasitoid’s searching behavior after it has already made its way to the plant [127].

Plant characteristics can modulate HIPV emission and plant volatile release fluctuates throughout the day [128], displaying the dynamic nature of volatile mosaics. Plant species release specific volatile mixtures upon attack by the identical herbivore species [129]. The level of volatile emission might vary between genotypes or cultivars of the same plant species [130], which could lead to different parasitism rates in the field [131]. Additional non-host herbivore infestations on the plant could change HIPV emission and, as a result, the attractiveness of the parasitoids happen [122]. The degree to which a nonhost herbivore’s assault modifies HIPV blends and affects parasitoids’ hunting behavior may differ depending on the species [132]. Therefore, an individual plant’s contribution to the volatile mosaic is determined by the attacking insects, both hosts and nonhosts.

**Figure 7:**
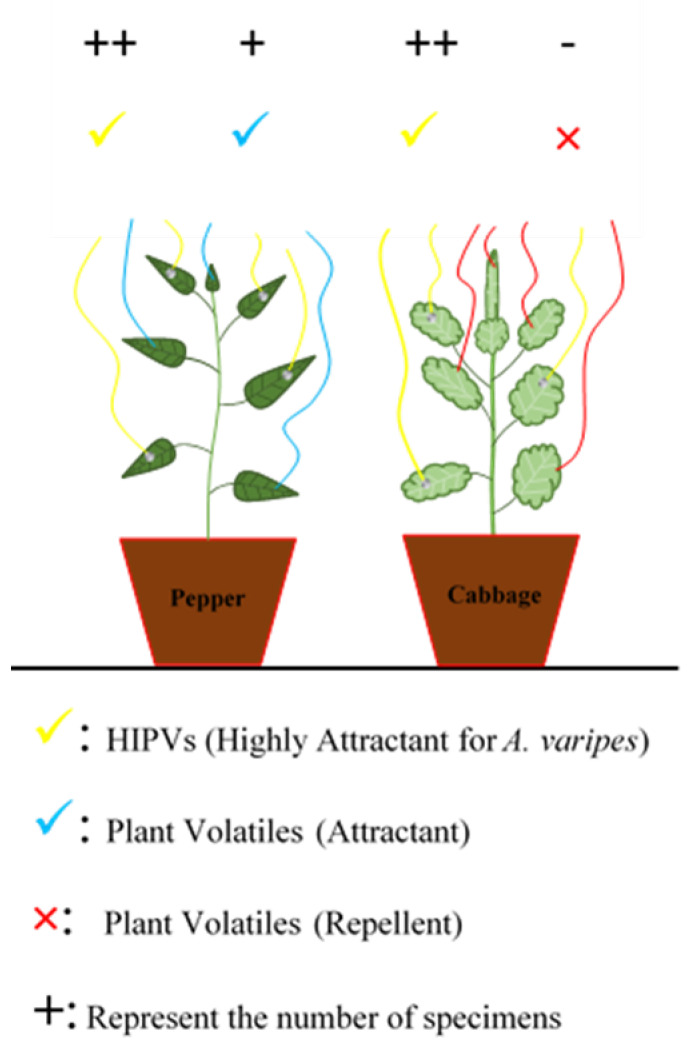
A schematic diagram to represent the volatile order emission and the response of the parasitoid *A. varipes* to these volatiles. Volatiles from healthy chili pepper attract more parasitoids than cabbage. The higher attraction was noted towards both plants after *Myzus persicae* damage however highest attraction was recorded towards chili pepper [7].

Furthermore, along with HIPVs, other pheromones such as chemicals released from herbivore byproducts (such as honeydew, frass, exuviae, defense secretions, mandibular gland secretions, etc.) and various stages of herbivores (eggs, larvae/nymphs, pupae, adults) are also used by natural enemies to choose oviposition and feeding sites [133]. For example, application of hydrocarbons, e.g., tricosane found in extracts of *Heliothis zea* Boddie (Lepidoptera: Noctuidae) moth scales, improved the ability of host location by the parasitoids *Microplitis croceipes* Cresson (Hymenoptera: Braconidae), *Trichogramma achaeae* Nagaraja and Nagarkatti (Hymenoptera: Trichogrammatidae), thus increasing the parasitism rate in the field [134,135]. In our latest study, we found that *Aphelinus varipes* Förster (Hymenoptera: Aphelinidae) wasp could differentiate between volatiles from non-identical plant species and were notably attracted towards HIPVs from chili pepper instead of other volatiles emitted from other plants and aphid/plant complexes (Figure 7) [7]. Studies definitively demonstrating increased Darwinian fitness, or more progeny in the following generation, in plants generating HIPVs are still missing, however [2].

### 3.2. Tritrophic Interaction in Plant to Plant Signaling

Information transfer within and between plants is one of the many hypothesized functions of HIPVs [136]. Numerous variables, such as plant species, genotype, age, herbivore species, attack severity, abiotic conditions, or combinations of these, might influence HIPV release at the plant scale. The earlier evidence for plant-to-plant signaling [1] was treated with skepticism, but it is now widely acknowledged that plants respond to their neighbors’ fluctuating stress signals [137]. Tritrophic interactions at the plant scale are specifically impacted by the interaction of biotic and abiotic stress factors [138].

Plant interactions lead to associational resistance. Biological control agents in the immediate environment can detect and process the volatile blend’s composition, which provides precise information about the physiological status of the plant [27]. These organisms also include neighboring plants and herbivores searching a host plant for egg deposition [139,140,141]. Healthy plants having herbivore-damaged neighbors are well known to acquire an increased level of resistance “associational resistance” to herbivores [139,142]. The resistance underlying plant–plant interactions is widely classified as active and passive mechanisms, both of which entail VOC transit between plants and are susceptible to environmental perturbation [143]. The active plant–plant interaction requires physiological change and a signal reception in receiver plants. Moreover, the passive interaction only entails chemical changes to the surface of the receiver plant as volatiles from an emitter plant adsorb to its surfaces [143]. Plant-emitted semi-volatile chemicals vaporize gradually around 20–25 °C and may thus linger on surfaces such as plant leaves [143]. The passive adsorption of arthropod-repellent semi-volatiles to neighboring vegetation may impart associational resistance, whereby a plant’s neighbors reduce damage caused by herbivores [144]. Adhered VOCs act as a repellent and provide protection even against fungal pathogen spores. These exogenous VOCs have opposite effects on herbivore and parasitoid behavior [145].

**Figure 8:**
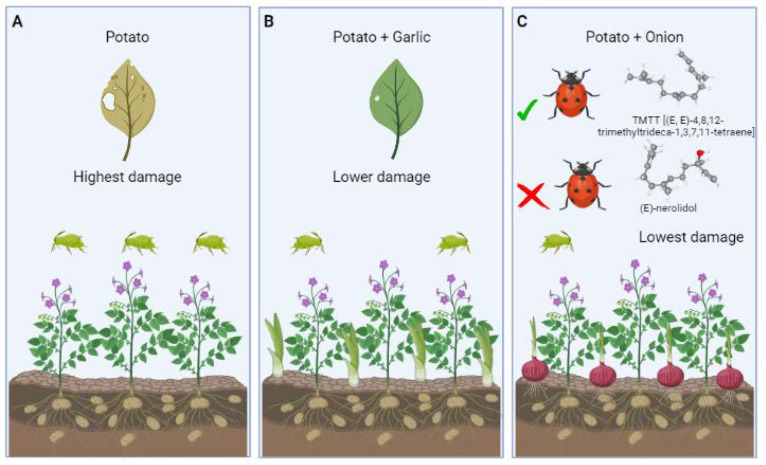
The impact of intercropping towards potato aphid (*Macrosiphum euphorbiae*) and its predator lady bird beetle (*Coccinella septempunctata*). (**A**): alone potato plant was highly susceptible to aphid attack and fall in highest damage category; (**B**): intercropping of potato alongside with garlic reduce damage of aphid and fall in lower damage category; (**C**): lowest aphid damage was recorded in potato + onion intercropping, meanwhile onion volatiles TMTT attract lady bird beetle however (*E*)-nerolidol repel lady bird beetle [19].

HIPVs play a role in triggering the stress response. A major discovery in the stress factors context was the release of methyl-jasmonate by stressed tomato plants, which prompts a defense response in nearby tomato plants [146]. Field evidence has since supported the communicative role of HIPVs in rapidly alerting undamaged tissues of incoming attack, hence overcoming vascular constraints [147]. The fact that surrounding plants use volatile signals is more likely due to eavesdropping than an intended warning by the emitting plant [137], even though warning of neighbouring kin can be an additional selective bonus [148]. In a field experiment, migration of potato aphids, *Macrosiphum euphorbiae* Thomas (Hemiptera: Aphididae) into potato, *Solanum tuberosum* L. (Solanum: Solanaceae) was significantly reduced by intercropping with the following three sequences, only potato plant: highest damage, potato with garlic, *Allium sativum* L. (Amaryllis: Amaryllidaceae) intercropping: lower damaged, potato with onion, *Allium cepa* L. (Asparagales: Alliaceae) intercropping: lowest damaged (Figure 8A–C) [148]. Furthermore, neighbor volatiles might variably influence natural enemies, as recently studied for ladybird, *Coccinella septempunctata* Linnaeus (Coleoptera: Coccinellidae) on potato exposed to onion volatiles: TMTT [(*E*, *E*)-4,8,12-trimethyltrideca-1,3,7,11-tetraene] was an attractant, whereas (*E*)-nerolidol acted as a repellent (Figure 8C) [30]. If the neighbor-emitted compounds are semi volatile in nature, the impact could be much stronger or more prolonged. Aphids have shown that behavioral responses to volatiles may only occur at higher concentrations. Comprehensive laboratory and field studies are lacking in intercropping environment to dig out the reason why aphid perform limited sensitivity; however, it is likely that aphids have trouble responding to lower amounts of volatiles because of their poor olfactory sensitivity.

### 3.3. Tritrophic Interactions at Landscape Scale

A series of behaviors is used by predators and parasitoids searching for their herbivorous prey, need to identify infected host plants in the diverse plant environment. This is a difficult endeavor since the host plants are a part of a highly heterogeneous semiochemicals environment made up of multiple herbivore populations and varied plant species, whose signals affect the ability to locate dependable kairomones during host or prey localization [149]. An “odor landscape” comprises many odor plumes (a blend of volatile compounds carried by the wind) that has been used by animals to navigate complicated dynamic sensory environment and make directional decisions for chemotactic orientation [150]. A recent review looked at how insects detect relevant or resource-indicating scent plumes throughout the olfactory landscape [151]. Foraging insects can follow one or more relevant odor plumes, changing from one to the next if one provides a more trustworthy signal (or points to a better resource), and they keep doing this until they locate the intended host plant [152]. In order to detect prospective infesting herbivores, locate the host plant within the plant community, and eventually choose and accept the target host or prey upon landing on the host plant, natural enemies while foraging depend on a variety of resource-indicating odors.

Landscapes are made up of a patchwork of different types of flora, all of which contain plants that can emit HIPV plumes. However, only a few studies have looked at how HIPVs affect parasitoid mobility at a landscape scale and how HIPVs from various patches affect parasitoid dispersal over a landscape [153]. It is difficult to trace parasitoid migration at broad geographical scales, because HIPV plumes are invisible and consequently difficult to measure in the field, making it challenging to investigate HIPVs in a landscape setting. In fact, rather than analyzing the movement patterns of individual parasitoids, the majority of landscape-scale studies infer parasitoid mobility using indirect techniques including examination of metapopulation structures [154]. Nevertheless, considering HIPV plumes may offer crucial insights into the dispersion and movement patterns of parasitoids at the landscape scale.

Applications of synthetic HIPVs-based lures in the field of chemical ecology continue to gain attention in biological control of insect pests. HIPVs based lures have been applied in several forms in the field however baited sticky traps are used widely. Methyl salicylate (MeSA: PredaLure) based HIPVs-lures have been significantly used to attract insect predators (Chrysopidae, Syrphidae, Hemerobiidae, Miridae, Coccinellidae and Anthocoridae) and parasitoids (Braconidae and Ichneumonidae) [2] in field crops (hops [155], soybean [73]) and fruit orchards (vineyard [156], apple pear and walnut [157,158]). HIPVs can selectively attract different natural enemies even at genus level to attack particular herbivore. For example, in fruit orchards high effectiveness of the blend made up of acetic acid, 2-phenylethanol and methyl salicylate were found in capturing various species of adult lacewings of the genus Chrysoperla (Chrysopidae) [157,158], but not to lacewings of the genus Pseudomallada [158]. Bio control agents are fine-tuned to volatiles released by plants upon attack and navigate upwind in these plumes to hunt prey [159]. Herbivore volatile fingerprints have commonly been shown to be species-specific [160]. One of the examples of HIPVs is geraniol mixed with 2- phenylethanol were able to capture Eupeodes (Syrphidae) in apple, pear and walnut orchards [157], but not attractive to Syrphidae in vineyard [158]. Moreover, both blends captured a relatively small number of Ichnemonoidea as well [158]. Comprehensive experimental techniques must be developed to determine if herbivore-induced emission is subsequently selective enough to be helpful to herbivore antagonists. The quantity of individual compounds may not be associated with their information value, necessitating a more extensive chemical examination of plant volatile mixtures that takes even small chemicals into account.

Field HIPV-based lures are generally applied in crops which also emit HIPV of interest, and this may improve interference with the successful location of lures by natural enemies [161]. Flint et al. [162] discovered that as cotton plants grew and emitted more compounds, the green predatory lacewing, *C. carnea*, became less attracted to synthetic caryophyllene, and thus detection of the lure thereby waned. However, *C. carnea* was not attracted to caryophyllene in wheat plants [163] for which it is one of the most prevalent volatile compounds [56]. In these circumstances, using concentrations above those of the target HIPVs in the plant field background odour or in particular blends may assist identify the pertinent concentration or blend composition that would best draw natural enemies to kairomone-based lures [164,165].

An alternative strategy for reducing the interaction between kairomone-based lure and volatile emission by target field crops could be to use an attractant HIPV in crop fields where HIPV is not emitted or only emitted in minor amounts to improve lure detection within the crop background odor. Phenylacetaldehyde baited field traps attracted 10–100-fold more predator *C. carnea* than un-baited traps when deployed in peach and cherry orchards [166]. Interestingly, the volatile profiles of peach and cherry plants hold minor amounts or no phenylacetaldehyde [167,168,169]. Therefore, unlike caryophyllene, it is expected that the predator *C. carnea* would be attracted to phenylacetaldehyde on cotton and wheat of which the headspace volatiles lack this compound [56,170,171,172].

The integration of many aspects that have been covered in this review is necessary to comprehend the interactions between arthropod natural enemies-herbivores-plants in a volatile-mosaic framework. The impression of the volatile mosaic may alter greatly based on the biocontrol agent size and style of movement. For instance, predator/parasitoids with low mobility may view volatile mosaics as fragmented, whereas with high dispersion capacity may not. Further research regarding the variables and mechanisms behind these natural enemies’ mobility and host hunting at the landscape scale needs to be better understood.

## 4. Conclusions and Future Perspectives

The discovery that HIPVs are major drivers of trophic interactions sparked a thriving field of highly multidisciplinary study. Since then, a lot of information has been developed, and it is becoming abundantly evident that inducible volatiles support a wide range of functions and ecological roles. Yet, many uncertainties exist, particularly about the mechanisms that are involved in the induction, evolution, release, and perception of the volatiles. An improved understanding of these mechanisms will also help us to better understand the ecological significance and evolution of HIPVs, as well as how we might better utilize them for crop protection. Technological advancements, such as molecular engineering and HIPV detection in real time, will enable precise modification, monitoring, and early identification of agricultural pests and diseases. In the long term, we believe that capitalizing on nature’s own innovations will pave the way for more sustainable, cost-effective agricultural production.

The HIPVs play a significant role in host plant–herbivore–natural enemy interactions and have the capacity to improve the effectiveness of host plant resistance and biological control for integrated pest management. However, there is a need to comprehend the ecological importance of HIPVs by integrating molecular and biochemical mechanisms in the production and recognize their ecological functions. Understanding such linkages will offer new options for future research on primary signaling pathways and their ecological repercussions in diverse natural and man-made ecosystems.

Longer-term studies of pest management considering HIPVs via promoting natural enemies are required because most research have been short in duration and consequently unable to disclose the effects of HIPVs concerning the changes in environment. In particular, it is yet unclear the impacts of genetically modified crops and pesticide use, shifts in land use in the surrounding landscape, and global warming. Moreover, agri environmental initiatives that pay farmers for stewardship activities provide chances to promote biological control by using HIPVs to encourage pest suppression. However, further study is needed to determine the impact of different HIPVs from plant taxa in a specific agri-ecosystem.

Another challenge is to attract and retain adequate natural enemies in crop fields, which are frequently suboptimal environments. To achieve this goal, the “Attract-and-Reward approach” could be achieved by incorporating attractive synthetically manufactured HIPVs with companion non-crop plants, which provide alternative resources to the targeted natural enemies [153,173]. Although often neglected, the spatial arrangement of HIPV dispensers and rewards within agricultural fields can have a significant impact on the foraging behavior and persistence of natural enemies, and therefore the efficacy of this pest control method. Furthermore, HIPVs could also play an important role in the “Push-and-Pull approach”, and research on the trade-offs and attractive/repellant stimuli interactions among multiple volatiles are urgently needed [174,175].

Last but not least, further research needs to be carried out to spot the volatile compounds that command the olfaction-directed behavior of insect pests and their natural enemies. Manipulation of such volatiles would attract the natural enemies of the crop pests for enhancing the effectiveness of bio-control agents for pest management. It will also be uttermost helpful to formulate strategies for developing pest resistant crop varieties with constitutive and induced resistance to insect pests.

## Figures and Tables

**Table 1:** Atmospheric impact on the lifetimes of selected herbivory induced volatile organic compounds and their interactions with substantial reactive air pollutants [113].

	BVOC	Lifetimes for Reaction with Oxidants			
HIPVs Compounds	Class	OH ^a^	O_3_ ^b^	NO_3_ ^c^	Reference
*cis*-/*trans*-Ocimene	Monoterpene	33 min	44 min	3 min	[116]
*β*-Phellandrene	Monoterpene	50 min	8.4 h	8 min	[116]
Linalool	Monoterpene	52 min	55 min	6 min	[116]
*β*-Caryophyllene	Sesquiterpene	42 min	2 min	3 min	[116]
*β*-Farnesene	Sesquiterpene	52 min	26 min	–	[121]
DMNT (4,8-dimethyl- ,3,7 nonatriene)	Homoterpene	40 min	60 min	3 min	I
TMTT (4,8,12-trimethyl- 1,3,7,11-tridecatetraene)	Homoterpene	30 min	30 min	2 min	I
*cis*-3-Hexenyl acetate	Green leaf volatile	1.8 h	7.3 h	4.5 h	[116]
*cis*-3-Hexen-1-ol	Green leaf volatile	1.3 h	6.2 h	4.1 h	[116]
*cis*-3-Hexenal	Green leaf volatile	11.2 day	3.0 h	–	[121]
Methyl salicylate	Aromatics	73.5 h	>9.8 year	–	[121]

BVOC: Biogenic volatile organic compound. Reference: I: Roger Atkinson + Jarmo K. Holopainen (Personal Communication). Different pollutant concentrations used in calculations: ^a^ Assumed OH radical concentration: 2.0 × 106 molecule cm^−3^ (0.074 pmol mol^−1^), 12 h daytime average; ^b^ Assumed O_3_ concentration: 7 × 1011 molecule cm^−3^ (26 nmol mol^−1^), 24 h average; ^c^ Assumed NO_3_ radical concentration: 2.5 × 108 molecule cm^−3^ (9.3 pmol mol^−1^), 12 h night time average.

## Data Availability

Not applicable.
